# Apelin in Reproductive Physiology and Pathology of Different Species: A Critical Review

**DOI:** 10.1155/2018/9170480

**Published:** 2018-06-06

**Authors:** Patrycja Kurowska, Alix Barbe, Marta Różycka, Justyna Chmielińska, Joelle Dupont, Agnieszka Rak

**Affiliations:** ^1^Department of Physiology and Toxicology of Reproduction, Institute of Zoology and Biomedical Research, Jagiellonian University in Krakow, 30-387 Krakow, Poland; ^2^INRA, Unité Physiologie de la Reproduction et des Comportements, 37-380 Nouzilly, France

## Abstract

Apelin has been isolated from the bovine stomach extracts as an endogenous ligand of the previously orphan receptor APJ. Expression of the apelinergic system (apelin and APJ) was described in many organs where pleiotropic effects like regulation of food intake, body weight, or cardiovascular and immune function were described. Recent studies have shown that apelin also plays an important role in the regulation of female and male reproduction. Some data showed that the gene and protein of apelin/APJ are expressed in the hypothalamic-pituitary-gonad (HPG) axis tissue. Thus, apelin is synthesized locally in the hypothalamus, pituitary, ovaries, and testis of many species and has autocrine and/or paracrine effects. Most research indicates that apelin has an inhibitory effect on gonadotropin secretion and participates in the direct regulation of steroidogenesis, cell proliferation, and apoptosis in gonads. The article summarizes also results of a series of recent studies on the effect of apelin on reproduction pathology, like polycystic ovarian syndrome, endometriosis, and ovarian cancer. Many of these pathologies are still in critical need of therapeutic intervention, and recent studies have found that apelin can be targets in reproductive pathological states.

## 1. Introduction

The hormonal interactions of the hypothalamic–pituitary–gonadal (HPG) axis are accountable for a proper physiology of both female and male reproduction. It is of importance to have knowledge of new regulators/hormones controlling reproduction. It is well known that adipose tissue is implicated in the secretion of several hormones such as adiponectin, resistin, leptin, visfatin, and apelin called adipokines “adipose tissue-derived hormones.” There is evidence that the increased production of adipokines might have a strong link to insulin resistance, metabolic syndrome, and obesity [1]. Apelin is a regulatory peptide, identified as an endogenous ligand of the apelin receptor named APJ [2]. Recently, the apelinergic (apelin and APJ) system was found in the HPG axis and apelin has been extensively described as a beneficial factor controlling reproduction both in females and in males. The intention of this paper is to review current knowledge concerning the expression of apelin/APJ in tissue of the HPG axis and physiological aspects of apelin on the physiology of both female and male reproduction. It will also describe apelin linked with reproduction dysfunctions like infertility, polycystic ovarian syndrome (PCOS), endometriosis, and ovarian cancer. Many of these pathologies are still in critical need of therapeutic intervention, and recent studies have found that apelin can be targets in pathological states. Therefore, apelin activity could be applied in the future in the treatment of many diseases of the reproductive system.

## 2. Apelin: Structure, Expression, and Function

### 2.1. Structure of Apelin

**Figure 1:**
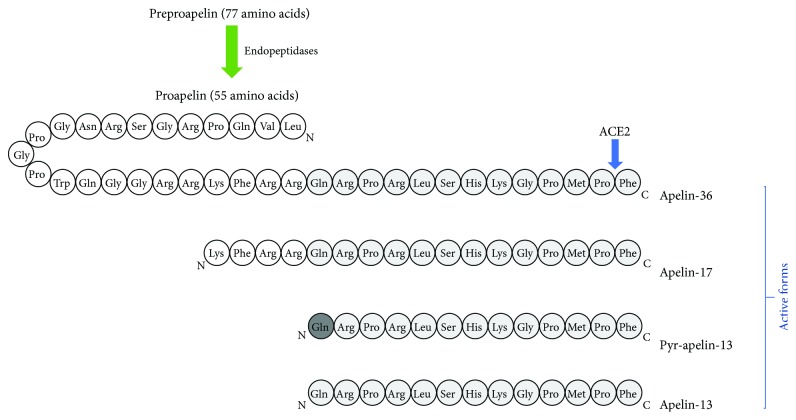
Amino acid sequence of native apelin and apelin isoform structure. ACE2: angiotensin I-converting enzyme 2.

Apelin has been isolated from the bovine stomach extracts as an endogenous ligand of the previously orphan receptor APJ (putative receptor protein related to the angiotensin receptor ATl), which is a G protein-coupled receptor [2]. Human apelin is encoded by the *APLN* gene located on chromosome Xq 25-26 [2]. This peptide has a 77-amino-acid preproapelin precursor and exists in multiple molecular forms with different biological activities. Native preproapelin, as a result of enzymatic hydrolysis, is transformed into active forms: apelin-36 (preproapelin-42–7), -17 (preproapelin 61–77), and -13 (preproapelin-65–77) and pyroglutamate-apelin-13 (pyr-apelin-13) (Figure 1) [2, 3]. Shorter forms of apelin (apelin-13) show much higher biological potency than longer forms do (apelin-36); thus, apelin-13 has been used for many different *in vitro* and *in vivo* experiments to investigate several physiological functions of apelin [2]. Additionally, pyr-apelin-13 and apelin-17 show a conserved binding to the angiotensin-converting enzyme 2 (ACE2) catalytic site and human ACE2 can cleave pyr-apelin-13 and apelin-17 [4]. Pyr-apelin-13 is a major isoform in human tissues, for example, in cardiac tissue from patients with coronary artery disease [5], and the plasma ranges from 7.7 to 23.3 pg/ml [6]. Moreover, pyr-apelin-13, apelin-13, and apelin-36 have similar efficacy and potency in cardiovascular tissues of humans [5].

The N-terminal region of apelin is rich in hydrophobic amino acids, indicating that these represent secretory signal sequences, while the C-terminal region has a sequence of 23 amino acids. It is conserved and critical for biological activity [2, 7]. Bovine, human, rat, and mouse preproapelin precursors have 76–95% homology. The endogenous form of these proteins is a dimer linked by a disulfide bond [6]. Mature forms of apelin do not have cysteine residues, and they are probably only monomeric proteins [7]. In order to bind apelin to its receptor, it is necessary to have a 13-amino-acid C terminus, which is observed in the in apelin-36 and pyr-apelin-13 [8].

### 2.2. Expression of Apelin

**Figure 2:**
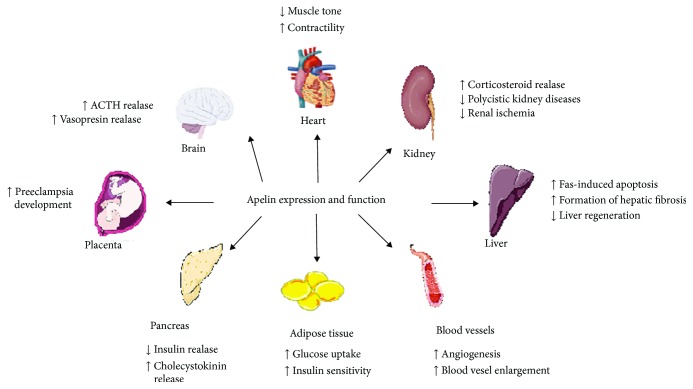
Apelin expression and function in the organism. ACTH: adrenocorticotropic hormone; PRL: prolactin; LH: luteinizing hormone; FSH: follicle-stimulating hormone.

Apelin expression (mRNA and protein) was detected in various tissues and organs such as stomach, brain, heart, lung, uterus, and ovary (Figure 2) [8–10]. Additionally, literature data also documented apelin localization in the endothelia of small arteries in many organs such as lung, spleen, liver, pancreas, and adipose tissues in rats [3, 11]. Expression of apelin increases during adipocyte differentiation, and its production is regulated by several factors such growth hormone (GH) or tumor necrosis factor (TNF-*α*) and insulin which increased apelin production by adipocytes [12].

### 2.3. Function of Apelin

The apelin signaling pathway plays a role in the central and peripheral regulation of the cardiovascular system, such as blood pressure and blood flow, in water and food intake, energy metabolism, and possibly immune function (Figure 2) [10, 13]. Apelin causes endothelium-dependent vasorelaxation by triggering the release of nitric oxide (NO), and it increases myocardial contractility [3, 14]. Moreover, it is reported that apelin is a potent angiogenic factor inducing endothelial cell proliferation, migration, and the development of blood vessels in an *in vivo* study [14, 15]. APJ mRNA expression was detected in areas of the brain critical for the control of fluid homeostasis, so apelin may play a role in the regulation of water balance [7]. Levels of apelin and APJ mRNA increase in white adipose tissue and plasma with obesity than in control subjects. However, obesity has to be associated with hyperinsulinemia [12, 16], so it may be the main cause for the rise in the expression of apelin. On the other hand, apelin inhibits insulin release [17]. Data of Heinonen et al. [16] showed a positive correlation between the level of apelin in plasma and the body mass index (BMI). Furthermore, research studies based on young females with eating disorders showed the highest level of apelin in the group of obese patients [17]. Apelin serum levels are related to the nutritional status and parallel insulin plasma levels in mice and humans [12, 18]. Furthermore, apelin plasma concentrations are increased in obese [16] and type 2 diabetic subjects [19] as well as in hyperinsulinemic obese mice [12]. In mice, apelin inhibited glucose-stimulated insulin secretion in pancreatic islets [20], suggesting a link with glucose homeostasis. Recently, a 14-day apelin treatment in mice was shown to regulate adiposity and to increase uncoupling protein expression [21], suggesting a role of apelin in energy metabolism. Literature data documented also that apelin has anti-inflammatory effects on the release of inflammatory mediators [22]. It also inhibits release of reactive oxygen species (ROS) in adipocytes and promotes an expression of antioxidative enzymes [23]. Additionally, apelin may play an important role in lymphatic tumor progression, because its overexpression was proved in rat malignant cells [24].

## 3. Characteristic of Apelin Receptor: APJ

### 3.1. Apelin Receptor (APJ)

This receptor is encoded by the APLNR (also known as AGTRL1, APJR, APJ, and FLJ90771) gene [25]. APJ is a class G protein-coupled receptor (GPCR) identified in 1993, and its structure shows high homology (40–50% in the transmembrane region) with angiotensin II receptor type AT1, but angiotensin II is unable to attach to this receptor [26]. The exact location of this gene was also determined for mice on chromosome 2E1 and for rats on chromosome 3q24 [27]. Both the structure and functioning of the human gene promoter APJ have not been fully understood [7]. APJ has a high (90%) similarity between human, rat, and mouse [28, 29] and about 50% between man and royal macaque, cow, frog, and zebrafish *Danio rerio* [7].

**Figure 3:**
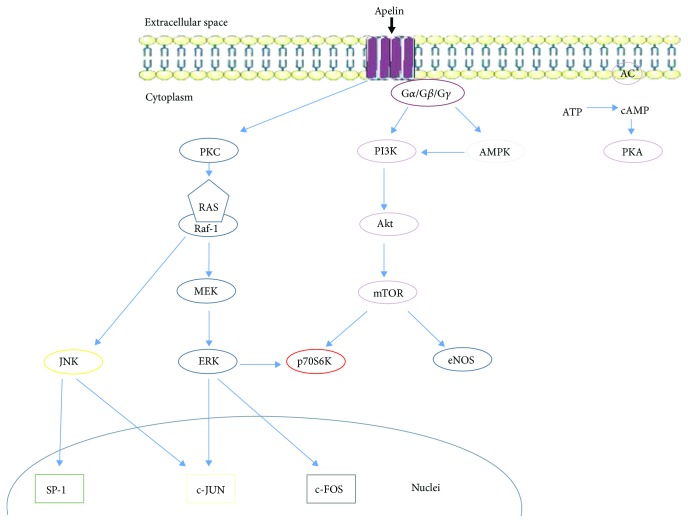
Pathways of apelin signaling after connection with APJ. PKC: protein kinase C: MEK-ERK activator kinase; ERK: extracellular signal-regulated kinase; JNK: c-Jun N-terminal kinases; SP-1: specificity protein 1; PI3K: phosphoinositide 3-kinase; Akt: protein kinase B; mTOR: mammalian target of rapamycin kinase; p70S6K: ribosomal S6 kinase; eNOS: endothelial NOS; AMPK: 5′-AMP-activated kinase: AC: adenyl cyclase; PKA: protein kinase A; ATP: adenosine triphosphate; cAMP: cyclic adenosine monophosphate; c-JUN: transcription factor c-JUN; c-FOS: transcription factor c-FOS.

APJ, due to the different affinity for various forms of apelin and cointeraction with different G (G*α*, G*β*, and G*γ*) proteins, interacts with activation of many signaling pathways [2] (Figure 3), thereby causing various effects in the body. In early experiments, apelin-13 has been observed to inhibit forskolin's stimulating effect on 3′,5′-cyclic adenosine monophosphate (cAMP) by binding APJ to the Gi/o protein [2]. These studies have been confirmed by Habata and coauthors [6], who proved that both apelin-13 and apelin-36 are not capable of generating calcium (Ca^2+^) mobilization in Chinese hamster ovary (CHO) cells. The different effects of both of these apelin isoforms are observed in neurons and in the human embryonic kidney cell line (HEK-293), where both isoforms increase Ca^2+^ levels [30]. APJ can also act via G*α*i1 and G*α*i2 proteins to inhibit adenylate cyclase in rats [31]. In turn, the CHO and the HEK-293 cell lines bind apelin with the APJ receptor via G*α*i2 and then consequently activate the extracellular signal-regulated kinase (ERK 1/2) pathway [32]. Additionally, apelin binding APJ activated phosphorylation of phosphoinositide 3-kinase (PI3K) and protein kinase B (Akt), which play an important role in cell proliferation or apoptosis. Apelin phosphorylates also the ribosomal S6 kinase (p70S6K) in human umbilical vein cells (HUVEC), thereby promoting the proliferation of these cells [31]. APJ signaling changes the level of ROS, so that apelin with APJ can stimulate catalase production and inhibit the production of hydrogen peroxide, thus protecting against cardiac hypertrophy [33]. In addition, apelin, by reducing ROS production and activating the actin kinase, protects mouse neurons from cell death [34]. One form of apelin, apelin-13, through kinase 5′AMP-activated kinase (AMPK) phosphorylation, lowers the process of mouse neuronal apoptosis after stroke. Studies on APJKO knockout mice have shown that apelin-13 by binding with APJ negatively regulates AMPK, which lowers the lipolysis process, the hydrolytic degradation of triglyceride in adipose tissue to fatty acids and glycerol [35, 36].

Gene and protein expression of APJ has been demonstrated in several tissues including the brain, ovary, kidney, pancreas, breast, and heart. Moreover, in humans, expression of APJ was high in the human brain and spleen and slightly lower in the ovary and placenta. In contrast, in the case of rat and mouse, the highest APJ expression was observed in the heart cells [7]. APJ expressions are regulated by many factors, for example, estrogens, insulin, cAMP, and CCAAT- (C/EBP-) binding protein, and strong brain stress significantly stimulates APJ secretion by adipose tissue cells [37].

### 3.2. ELABELA/Toddler as a Ligand of APJ

The recent discovery of a new endogenous peptide ligand for APJ, currently known as both Toddler [38] and ELABELA [39], followed screens to discover signals regulating early development. Although characterized in zebrafish, a high degree of conservation of the ELABELA/Toddler gene in vertebrate species including humans implies likelihood of similar importance in human development, but this has yet to be shown. Like apelin, this peptide exists in multiple endogenous isoforms [40]. ELABELA/Toddler signaling is motogenic, and its absence or overproduction reduces the movement of mesendodermal zebrafish cells during gastrulation, inhibiting proper development [38]. Moreover, in ELABELA/Toddler KO knockout zebrafish, the cells of the endoderm have impaired differentiation potential and embryos exhibit stunted or completely absent heart development. This mirrors the phenotype observed in targeted deletion of APJ (APJKO) embryos [39]. Apelin KO embryos, on the other hand, do not have this phenotype. Systemic administration of ELABELA/Toddler in ELABELA/Toddler KO zebrafish rescues the otherwise aberrant phenotype [38].

Receptor activation studies revealed that the zebrafish Toddler-21 peptide acts by binding APJ and inducing receptor internalization [38]. Moreover, the expression profiles of ELABELA/Toddler and apelin differ during zebrafish development [38]. In particular, during gastrulation ELABELA/Toddler is highly expressed, whereas apelin expression remains low. Following this period, however, ELABELA/Toddler expression drops sharply and apelin levels begin to rise steadily. All these findings indicate that ELABELA/Toddler is a developmentally critical APJ ligand whose signaling behavior differs significantly from that of apelin. The exact intracellular signaling mechanism(s) of ELABELA/Toddler remains unknown. ELABELA/Toddler by activated G protein- and *β*-arrestin-dependent pathways acts in the human heart. Moreover, apelin acting on cardiac contractility and vasodilatation in *in vitro* experiments in rat heart [41]. Another team discovered that ELABELA/Toddler increases cardiac contractility in an ERK1/2-dependent manner in adult rat hearts [42].

## 4. Physiology and Pathology of Apelin in the Hypothalamus–Pituitary Axis

### 4.1. Expression and Effect of Apelin on the Hypothalamus–Pituitary–Axis

The central nervous system, especially in the hypothalamus and pituitary, contains primary sites of apelin action. The apelinergic neurons were firstly observed in the central nervous system of rats using the immunohistochemistry method [43], indicating the topographical distribution of apelinergic neurons suggesting multiple roles for apelin in the control of behaviors, pituitary hormone release, and circadian rhythms. Apelin and APJ gene expression was observed in the hypothalamic supraoptic nucleus and in the magnocellular and parvocellular parts of the paraventricular nucleus (PVN) in rats [43]. In the hypothalamus, apelinergic nerve fibers were detected in the periventricular, suprachiasmatic, ventromedial, dorsomedial, nucleic, and retrochiasmatic areas. The immunofluorescence method shows that apelin-immunoreactive neuronal cell bodies were localized throughout the rostrocaudal extent of the mouse activity-regulated cytoskeleton-associated protein (Arc). Moreover, apelin localized with proopiomelanocortin (POMC) and weakly with neuropeptide Y (NPY). By immunohistochemistry using in situ hybridization, APJ is present in Arc POMC neurons. Apelin/APJ mRNA was also detected in the anterior and posterior pituitary and in intermediate lobes of the rat pituitary [29]. Moreover, Reaux et al. [43] using immunofluorescence staining discovered that apelin is coexpressed in the anterior pituitary with corticotrophs and somatotrophs using rats as model.

The hypothalamic localization of apelin fibers and receptors suggests an involvement of apelin in the control of hormone release [44]. In an ex vivo perifusion system of rat anterior pituitaries, apelin-17 significantly increased basal adrenocorticotropic hormone (ACTH) release [45]. Moreover, results of the perifusion technique for hypothalamic explants have been demonstrated that apelin-17 increased *α*-melanocyte-stimulating hormone (*α*-MSH) release, suggesting that apelin released somatodendritically or axonally from POMC neurons may stimulate *α*-MSH release in an autocrine manner [46]. In the hypothalamus, apelin may be involved also in food intake; in rats, apelin-13 intracerebroventricular (icv) injection increased food intake by inhibited cocaine- and amphetamine-regulated transcript (CART) mRNA expression and serotonin secretion and by increased orexin mRNA expression in the hypothalamus [47]. Chronic icv infusion of apelin in the mouse hypothalamus increased also the expression of proinflammatory factors, associated with higher levels of interleukin-1 beta in plasma [48]. Apelin-13 in the PVN increased c-*Fos* expression [49] and secretion of both plasma ACTH and corticosterone (CORT) [50, 51]. Moreover, icv administration of pyr-apelin-13 was used to indicate where the postranslation modification occurs and showed apelin-13 decreasing prolactin (PRL), luteinizing hormone (LH), and follicle-stimulating hormone (FSH) levels [50]. An *in vitro* study documented that apelin-13 increased the release of corticotropin-releasing hormone (CRH) and vasopressin (AVP) from hypothalamic explants, with no effect on NPY release [44, 50], suggesting that apelin may play an important role in the hypothalamic regulation of water intake and endocrine axes. Newson et al. [52] using APJ KO mice had established a role for APJ in the integration of neuroendocrine responses to acute stress and had demonstrated a gender-specific function of apelin in peripheral immune activation of the hypothalamus–pituitary–adrenal axis [52]. Moreover, Tobin et al. [53] documented that apelin-13 administration onto the hypothalamic supraoptic nucleus increased the firing rates of vasopressin cells but had no effect on the firing rate of oxytocin neurons, suggesting a local autocrine feedback action of apelin on magnocellular vasopressin neurons.

An icv administration of apelin-13 produced a dose- and time-related antinociceptive effect; this effect was significantly antagonized by the APJ receptor antagonist apelin-13, indicating an APJ receptor-mediated mechanism [54]. Apelin-13 is also involved in the autophagy suppression of neural cells; thus, it attenuates traumatic brain injury [55]. In lactating rats, apelin modulates the activity of oxytocin neurons; the activity is inhibited by a direct action of the apelin on its receptor, expressed by these neurons [56].

## 5. Physiology and Pathology of Apelin in the Ovary

### 5.1. Expression and Function of Apelin/APJ in the Ovary

The apelinergic system was found in the ovary of many species like bovine, rhesus monkey, porcine, and human (Table 1) [13, 57–61]. Shimizu et al. [62] demonstrated that in bovine follicles the expression of apelin mRNA was not found in granulosa cells (Gc), while the APJ gene was increased in Gc of estrogen-inactive dominant follicles. Additionally, the expression of apelin mRNA increased in theca cells (Tc) of estrogen-inactive dominant follicles but APJ expression in Tc increased with follicle growth [62]. *In vitro* experiments of bovine ovarian cells showed that several factors regulated apelin/APJ expression; for example, progesterone (P4) and FSH stimulated the expression of APJ mRNA in the cultured Gc, while LH induced the expression of apelin and APJ in cultured Tc [62]. In the next study, Schilffarth et al. [13] observed that in the bovine ovary, the expression level of apelin during the oestrous cycle was significantly higher compared to the one during pregnancy. Moreover, apelin mRNA was high during the cycle and decreased after corpus luteum (CL) regression, while in ovarian follicles the expression of apelin/APJ was significantly upregulated in follicles with an estradiol (E2) concentration of more than 5 ng/ml, suggesting that the apelin/APJ system is involved in the mechanism regulating angiogenesis during follicle maturation as well as during CL formation and function in the bovine ovary [13]. Our last data demonstrated that the expression of both apelin and APJ in bovine granulosa and oocytes significantly increased with ovarian follicle size whereas it was similar in theca interstitial cells [59]. Furthermore, *in vitro* experiments showed that insulin-like factor I (IGF1) increased apelin expression, whereas it decreased the mRNA expression of APJ [59]. In the porcine ovary, apelin concentration in the follicular fluid and expression of both apelin and APJ increased with follicular growth; the greatest values were found in large follicles [61]. Immunohistochemistry revealed the positive staining for apelin and APJ in membranes of porcine Gc, than in Tc; additionally, a strong expression of apelin in oocytes and APJ in the zona pellucida was observed [61]. Similar as in bovine CL, our data also documented that in porcine CL, apelin/APJ is dependent on the CL growth and development phase; apelin expression was similar in early and middle CL and then decreased in regressing CL [63]. Moreover, localization of apelin was found in the cytoplasm of luteal cells in all stages of CL development, while the strongest APJ staining was found in middle cells [63]. Roche et al. [58] demonstrated apelin and APJ at the gene and protein levels also in human ovarian cells and granulosa cell lines (KGN). These authors demonstrated higher immunolocalization of APJ in human Gc, cumulus, and oocyte as compared to Tc. The high expression is also demonstrated in primary, medium, and mature follicles; apelin/APJ is expressed in the cytoplasm and nuclei of Gc [58].

The presence of apelin/APJ (Table 1) in various ovarian cells and its change during ovarian follicles and CL development suggests a potential role of apelin in the control of several aspects of ovarian cell function such as folliculogenesis, steroid hormone secretion, proliferation, or apoptosis. *In vitro* studies indicate that apelin may directly regulate steroidogenesis in ovarian cells. Apelin by activation of the APJ receptor causes a statistically significant increase in P4 and E2 secretion and 3*β*-hydroxysteroid dehydrogenase/*Δ*^5–4^ isomerase (3*β*HSD) protein level both in primary cell cultures and in IGFI-induced human and porcine ovarian cells [58, 59, 61]. As a molecular mechanism of apelin action on the steroid synthesis process authors considered activation of the serine–threonine kinase, mitogen activated protein kinase (MAPK3) and AMPK kinase pathways [58]. Similar results have been obtained in *in vitro* studies of bovine ovarian cells, which show that apelin stimulates P4 production and proliferation of these cells by activating Akt kinase [59]. In addition, the authors demonstrated an inhibitory effect of apelin on the *in vitro* maturation of bovine oocytes and the release of P4 by cumulus cells, indicating the direct role of this adipokine in the maturation of oocytes. Shuang et al. [64] showed that apelin stimulates proliferation and inhibits the process of apoptosis in rat Gc by activating the Akt kinase pathway. In addition, Shimizu et al. [62] suggest involvement of apelin in follicular atresia induced by Gc apoptosis during bovine follicular because they have demonstrated high expression of the APJ receptor in atretic bovine follicles.

Apelin is also a regulator of the CL luteolysis process [57]. In the middle CL (sensitive to PGF2*α*), a transient increase in blood flow associated with the stimulation of endothelium nitric oxide (eNOS) was observed, which is the first signal that initiates luteolysis [65]. Apelin activates the eNOS pathway through stimulation of nitric oxide production, resulting in the expansion of blood vessels [3]. Another mechanism to explain the luteolytic effect of apelin is CL apoptosis. Apelin is one of the factors that slow down the process of ovarian apoptosis. On the other hand, apelin induces the expression of the antiapoptotic B-cell lymphoma 2 (Bcl-2) protein, while decreasing proapoptotic Bax production further blocks the release of cytochrome c and activates the caspase-3 apoptosis executive enzyme resulting in apoptosis suppression in osteoblast cells [66].

### 5.2. Apelin and Ovarian Pathology

**Figure 4:**
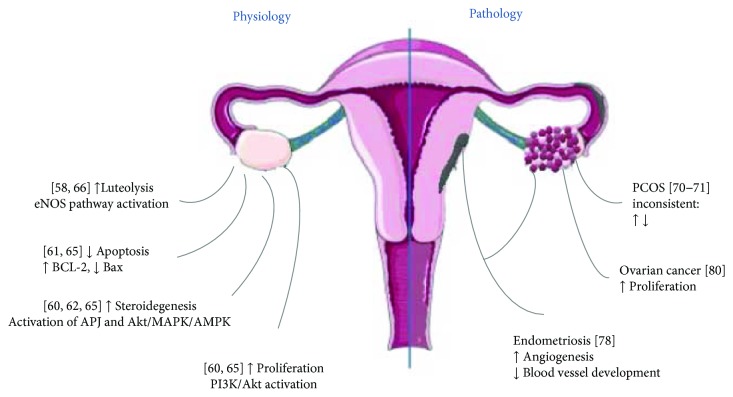
Apelin effect on ovarian physiology and pathology. eNOS: endothelium nitric oxide; Bcl-2: B-cell lymphoma 2; APJ: apelin receptor; Akt: protein kinase B; MAPK: mitogen-activated protein kinases; AMPK: 5′AMP-activated kinase; PI3K: phosphoinositide 3-kinase; PCOS: polycystic ovary syndrome.

PCOS is the most common cause of infertility due to lack of ovulation. This syndrome was first described by Stein and Leventhal in 1935. They described women with excessive hair, obesity, and ovaries covered with cysts. It is the main endocrinopathy of reproductive-age women. PCOS also binds to insulin resistance, which results in hyperinsulinism, which affects the production of androgens by the ovaries and adrenal glands. There are also changes in the lipid and carbohydrate economy, which in turn leads to diabetes type 2 and cardiovascular and biliary tract diseases. Increased risk of endometrial cancer and diabetic pregnancies, preeclampsia during pregnancy, or venous thrombosis are also symptoms of this condition [67]. Genetic factors responsible for PCOS pathogenesis are mutations in the genes responsible for steroid hormone synthesis, regulation of gonadotropins, and those associated with the pathway for weight regulation. Environmental factors can also be classified as obesity, occurring in 50% of patients, resulting in disorders of implantation, cycle, ovulation, and miscarriage [68]. The results of Roche's et al. [58] data compared the expression of apelin and APJ in Gc from obese or nonobese patients with or without PCOS. They observed that apelin and APJ mRNA expression is increased in PCOS patients, and it was higher in obese patients [58], suggesting the role of apelin as a marker of PCOS pathogenesis (Figure 4). Moreover, higher levels of apelin-13 in follicular fluid in obese women compared to nonobese women in both the PCOS and non-PCOS groups was observed [58]. However, the published data comparison of serum apelin levels in PCOS and non-PCOS women is inconclusive. Some authors point to its considerable elevation in serum PCOS [69–74]. Data of Sun et al. [72] indicated a weight-dependent increase in the concentration of apelin in obese women with PCOS compared to PCOS-deficient women. Apelin was found to be higher in PCOS patients by Gören et al. [70] but without a significant correlation with homeostatic model assessment (HOMA-IR). Olszanecka-Glinianowicz et al. [73] reported an inverse association between apelin and glucose, insulin, and HOMA-IR values, supporting the role of apelin in the regulation of insulin sensitivity. Apelin levels were higher in nonobese PCOS patients, suggesting a compensatory mechanism for metabolic consequences of insulin resistance. Comparative results of studies showing lower serum apelin levels in PCOS have been obtained by several authors [73–76]. Different from Cekmez et al.'s study [69], lower serum concentrations of apelin were found in PCOS subjects by Altinkaya et al. [75] with a positive correlation with BMI, insulin, HOMA-IR, triglyceride, and free testosterone, speculating that apelin can be used as a marker for insulin sensitivity. Conversely, Sun et al. [72] observed a significantly enhanced apelin concentration in PCOS patients with a positive association with BMI and HOMA-IR; treatment with drospirenone–ethinylestradiol plus metformin improved insulin resistance and apelin levels. Discrepant findings among the published studies may be attributed to the differences in ethnicity, age, study design, sample size, genetic characteristics of populations, and assessment methodology, defining PCOS definitions; the difference in the test was used to analyze the concentration of different apelin isoforms.

Another ovarian pathology that has been recently linked to apelin action is endometriosis. Endometriosis is a disease which is characterized by the survival and growth of endometrial tissue outside the uterus primarily in the pelvic area. It is one of the most common gynecological diseases with up to 10% of women in the USA suffering from its symptoms which include infertility and severe pelvic pain [77]. This disease is highly estrogen-dependent and is accompanied by a major inflammatory response. Apart from surgical removal of endometriotic lesions, the main therapeutic approach is continuous treatment with progestins to inhibit the proliferation of this ectopic tissue which is not always effective [77]. Therefore, investigation of steroid hormone signaling in this disease is critical to identifying new therapeutic targets. Apelin might be a factor playing a role in the endometrial regeneration via angiogenesis. Ozkan et al. [77] using the immunohistochemistry method and immunoassay detected apelin in the eutopic and ectopic endometrium of women with or/and without endometriosis. Apelin concentrations increased during the secretory phase and decreased during proliferative phases of eutopic and ectopic endometrial tissue. Moreover, the higher immunoreactivity of apelin was observed in the endometrium in the secretory phase and in glandular cells of both eutopic and ectopic endometrial tissues, suggesting that increased local apelin concentration may indicate a paracrine function on the endometrium [77]. Additionally, apelin causes endothelium-dependent vasorelaxation by triggering the release of nitric oxide and is a potent angiogenic factor inducing endothelial cell (EC) migration, proliferation, and blood vessel *in vivo* development, indicating its effects as a chemoattractant for endothelial cell growth [77].

Recent data indicate the relationship between apelin and ovarian cancer. Ovarian tumors, the second most common type of gynecological malignancy [78], are heterogeneous neoplasms classified into three major categories, namely, epithelial ovarian tumors, sex cord-stromal tumors (e.g., granulosa cell tumors), and germ cell tumors. Epithelial tumors account for 80% to 90% of ovarian malignancies, whereas Gc tumors account for 1% to 2% of ovarian malignancies in the USA and Europe. Data of Hoffmann et al. [79] documented the expression of apelin/APJ in different ovarian cell lines; they observed that the APJ expression level was higher in epithelial cancer cells than in Gc tumor, whereas the reverse was true for apelin expression and secretion. Additionally, these data indicate that apelin stimulated OVCAR-3 cell proliferation and suggest its mitogenic action in ovarian epithelial cancer cells. Furthermore, recent studies report that apelin stimulates cancer cell migration in the lung, oral cavity, and colon [80, 81].

## 6. Physiology and Pathology of Apelin in the Testis

To our knowledge, there is one published data demonstrating the effect of apelin on male reproduction. Infusion of apelin-13 in male rats significantly suppressed LH release compared with the vehicle values, while levels of FSH did not significantly differ among the groups [82]. Furthermore, serum testosterone levels in the apelin-13 group were statistically lower than in the control group; histological examination showed that infusion of apelin-13 significantly decreased the number of Leydig cells, suggesting that apelin may play a role in the central regulation and decrease testosterone release by suppressing LH secretion. Finally, these authors concluded that the agonist of APJ may be a useful drug for pharmaceuticals in the treatment of male infertility [82].

## 7. Summary and Conclusion

In summary, the apelinergic (apelin and APJ) system was found in the hypothalamus, pituitary, ovaries, and testis of many species and has autocrine and/or paracrine effects on control reproduction both in female and in male regulation of their physiology. Most research indicates that apelin has an inhibitory effect on gonadotropin and PRL secretion in females, while in male rats, an inhibitory effect of apelin on LH and testosterone was observed in *in vivo* experiments. Apelin also participates in the direct regulation of ovarian physiology; it was clearly documented that apelin has a stimulatory effect on steroidogenesis and proliferation but an inhibitory action on cell apoptosis by activation on several kinase pathways such as AMPK, ERK, and Akt. Based on available data, we speculated that apelin has a connection with such dysfunctions like PCOS, endometriosis, and mitogenic action in ovarian cancer. Many of these pathologies are still in critical need of therapeutic intervention, and recent studies have found that apelin can be a target in pathological states. Therefore, apelin activity could be applied in the future in the treatment of many diseases of the reproductive system.

## Figures and Tables

**Table 1:** Apelin/APJ expression in the ovary of many species and direct effects of apelin on ovarian function.

Species	Apelin/APJ expression				References	Effect of apelin on ovarian function			Apelin doses	References
	Granulosa	Theca	Oocyte	CL		Steroidogenesis	Proliferation	Apoptosis		
Human	+/+	+/+	+/+	ns	[58]	↑P4, ↑E2	ns	↓	10^−9^ M	[58]
Pig	+/+	+/+	+/+	+/+	[61]	↑P4, ↑E2	ns	ns	0.02, 0.2, 2, and 20 ng/ml	[61]
Bovine	−/+	+/+	+/+	+/+	[13, 57, 59, 62]	↑P4	↑	ns	10^−9^ M, 10^−8^ M, and 10^−6^ M	[59]
Rat	ns	ns	ns	ns	—	ns	↑	↓	10^–8^ mol/l	[64]
Rhesus monkey	+/+	+/+	ns	+/+	[60]	ns	ns	ns	ns	—

+: present; − does not exist; ns: no study; ↑: increase; ↓: decrease; P4: progesterone; E2: estradiol.
